# Determining the Prioritization of Behavior Change Techniques for Long-Term Stroke Rehabilitation: Delphi Survey Study

**DOI:** 10.2196/59172

**Published:** 2025-04-07

**Authors:** Agata Ewa Wróbel, Philip Cash, Anja Maier, John Paulin Hansen

**Affiliations:** 1 Department of Technology, Management and Economics, DTU Technical University of Denmark, Lyngby, Denmark; 2 Design School, Northumbria University, Newcastle, United Kingdom; 3 Department of Design, Manufacturing and Engineering Management, University of Strathclyde, Glasgow, United Kingdom

**Keywords:** behavior change, behavior change techniques, BCT, neuroscience, neurology, stroke, rehabilitation, adherence, Delphi method, Delphi, intervention design, intervention mapping

## Abstract

**Background:**

Stroke results in both physical disability and psychological distress. The impact can be minimized through rehabilitation, but it is a long-term process, making it difficult for patients to adhere to treatment. Thus, a better understanding of long-term behavior change interventions for patients with stroke is needed as well as how such interventions can support not only rehabilitation of motoric functions but also mental well-being.

**Objective:**

The aim of this study is to understand both the most important behavior change technique (BCT) clusters for long-term stroke rehabilitation in general as well as which are most relevant for each aspect of stroke rehabilitation: behavioral, cognitive, and emotional.

**Methods:**

We applied the 16 BCT clusters. The study used a 2-round Delphi survey, as reliable consensus was obtained among a group of 12 international experts. Experts represented three main backgrounds involved in behavioral intervention in the health context: (1) specialists in behavioral science (n=4), (2) behavioral designers (n=4), and (3) expert health care professionals (n=4). Experts were brought together in this way for the first time. In the first round, web-based questionnaires were used to collect data from the experts. This was followed by a personalized second round. Consensus was determined by statistically aggregating the responses and evaluating IQR and percentage consensus. BCT clusters reaching consensus (IQR ≤1 and percentage ≥50%) were then ranked.

**Results:**

In total, 12 of 16 BCT clusters reached consensus for general importance in stroke rehabilitation, with 11, 9, and 6 BCT clusters achieving consensus for, respectively, the behavioral, cognitive, and emotional aspects of rehabilitation. The overall most relevant BCT clusters were repetition and substitution, social support, feedback and monitoring, and self-belief, with similar outcomes for behavioral and cognitive rehabilitation. For emotional rehabilitation, social support and identity were emphasized. The least relevant BCT clusters were natural consequences, covert learning, and comparison of behavior.

**Conclusions:**

This expert panel study using a 2-round Delphi survey ranked the importance of BCT clusters for long-term stroke rehabilitation. The process yielded a number of novel insights highlighting differences in importance between general rehabilitation and that specifically focused on the behavioral, cognitive, and emotional aspects of stroke recovery. This provides a first but important step toward unlocking the prioritization of BCT clusters for long-term intervention contexts such as stroke rehabilitation and enables effective intervention mapping addressing long-term behavior change and treatment adherence.

## Introduction

### Background

The World Health Organization defines stroke as “(...) rapidly developed clinical signs of focal or global disturbance of cerebral function, lasting more than 24 hours or until death, with no apparent non-vascular cause” [1]. It is a serious health problem and one of the most common causes of death and acquired disability among adults [2], having “(...) the greatest disabling impact of any chronic disease” [3]. Further, only a small proportion of patients die in the acute stroke phase, leaving the majority with moderate to severe disability that can be mitigated through early and sustained rehabilitation interventions [4]. It is estimated that the number of people living with stroke will increase by 27% in the European Union alone between 2017 and 2047 [5]. As such, stroke has a considerable socioeconomic impact, affecting not only patients themselves but also their families, caretakers, medical professionals, hospitals, policy makers, and the whole health care system in the long term [6]. This places a critical focus on effective poststroke treatment and rehabilitation [7] not least for making health care interventions usable, safe, and effective.

Patients with stroke experience both physical disability and psychological changes, including emotional, behavioral, and cognitive impairments [3]. Issues, including depression, apathy, anxiety, posttraumatic stress, and anger, among others [3], are prevalent in patients with stroke. These pose significant barriers to rehabilitation by negatively impacting long-term adherence to exercises, hindering treatment processes, and decreasing the chance of patient recovery [8,9]. This places a priority on positively shaping patient behavior change in the long term. Yet, Teasell et al [10] highlight that over two-thirds of interventions in this context focus on improving motor functions, while less than 6% dealt with psychosocial issues. As such, there is a need to highlight the relevance of behavior change techniques (BCTs) in addressing psychosocial issues. However, general discussions of BCTs typically focus on interventions framed in the short term [11]. For example, Liu et al [12] highlight how a single reward can be effective in fostering short-term sign-up behavior, but additional strategies and research are needed to understand how best to maintain engagement. This uncovers a gap between current stroke rehabilitation practice and the potential of BCTs, as it leaves critical questions as to which BCTs should be prioritized when supporting the long-term treatment adherence required. Further, this reinforces the relevance and urgency of addressing long-term behavior change and treatment adherence specifically for stroke rehabilitation.

Together, these challenges reveal a critical disconnect between current stroke rehabilitation interventions and more general guidance on BCT use [13]. Thus, this work focuses on resolving this disconnect by examining how BCTs should be prioritized for stroke rehabilitation with a multifaceted approach that distinguishes between motor rehabilitation (“general rehabilitation” in our terms) and psychosocial rehabilitation (addressed separately as “behavioral,” “cognitive,” and “emotional” aspects). As such, the aim of this study is to understand both the most important BCT clusters for long-term stroke rehabilitation in general as well as which are most relevant for each aspect of stroke rehabilitation: behavioral, cognitive, and emotional.

### Patient Adherence to Treatment

Poor adherence to treatment is a common challenge across health care domains [8]. In physiotherapy, up to 65% (with some sources claiming even 70%) of patients are nonadherent or only partially adherent to home treatment and exercises [8,14]. This high level of nonadherence (noncompliance) has an adverse effect on the cost and effectiveness of rehabilitation [8,15]. Several studies to date have explored the reasons for patient nonadherence to home treatment and exercises [8,14,16,17]. In their review, Jack et al [8] found strong support for the following barriers to treatment adherence: “low levels of physical activity at baseline or in previous weeks, low in-treatment adherence with exercise, low self-efficacy, depression, anxiety, helplessness, poor social support or activity, greater perceived number of barriers to exercise, and increased pain levels during exercise.” However, while the importance of identifying and acknowledging the above during patient assessment has been emphasized, few authors offer concrete solutions to address adherence.

Two notable exceptions to this are Campbell et al [15] and Bonnechre et al [16]. Campbell et al [15] note the importance of physiotherapist’s presence and contact with the patient as well as including patients as partners in planning therapy; and Bonnechre et al [16] stress the positive effect of patient supervision and suggest that, for example, telerehabilitation or other monitoring systems could be beneficial. However, when compared to the scope of relevant BCTs [18], these examples primarily serve to highlight the untapped potential of behavior changing interventions in support of long-term stroke rehabilitation. First, they are not specific to stroke rehabilitation; hence, they do not address some key issues that patients with stroke deal with compared to other conditions, such as simultaneous impairment of both motoric and cognitive functions. This makes the rehabilitation process more complex and increases the risk of developing other conditions such as depression and anxiety. Second, many approaches to improve patient adherence lack individualization, which is crucial as every patient is “(...) structurally, chemically, and emotionally different” [19]. This is especially true in the case of stroke, which can occur at different ages (despite being more prevalent in older age groups), be located in different regions of the brain with different functional consequences, and yield a range of effects, from mild to very severe [5]. Therefore, potential interventions must be considered in terms of long-term feasibility, as well as suitability for home treatment and rehabilitation, with minimum assistance of third persons. Each of these challenges points to potential mechanisms found in BCTs.

### BCTs in Health Care

BCTs used as the basis for health care interventions such as education, training, or facilitating behavior through design, have been successfully applied to drive changes in health care professionals’ practice [20,21], patient treatment and rehabilitation [22], disease prevention [23], or self-management [24]. At the same time, while the topic has been studied extensively by both behavioral psychologists [18] and designers [25,26], the differentiation between short- and long-term interventions has been little discussed. This poses a notable issue for rehabilitation, as it is widely acknowledged that sustaining long-term change introduces specific behavioral and design challenges [11,27,28].

A general approach to designing health care interventions suggests the following steps: “(...) identifying barriers, selecting intervention components, using theory, and engaging end-users” [21], linking development to feasibility, evaluation, and implementation [29,30]. In rehabilitation specifically, particular attention is given to the rehabilitation provider, who acts as a facilitator and enabler in driving health behavior change [31]. Nieuwenhuijsen et al [31] also highlight the need to understand a person’s context and environmental factors, as these can become facilitators or barriers to change. While these considerations are essential, guidance on how to approach long-term change or prioritize BCTs in this context is missing. This is particularly relevant in the case of stroke. This is because after the first few months of intense treatment, rehabilitation can take several months or even years in a home setting. Further, due to the complexity and scope of possible BCTs, prioritization efforts are critical to their understanding and application [18,32]. Here, one of the most widely accepted taxonomies of BCTs is provided by Michie et al [18]. Within the taxonomy of Michie et al [18], there are 16 clusters, with 93 individual BCTs, presenting a huge scope for intervention. Therefore, there is an urgent need to better understand which BCT clusters are most relevant to interventions for long-term contexts such as stroke rehabilitation.

### Research Question

Bringing the literature together, there is a need to understanding both the most important BCT clusters for long-term stroke rehabilitation in general as well as which are most relevant for each aspect of stroke rehabilitation: behavioral, cognitive, and emotional, that is, supporting not only motoric and cognitive function but also mental well-being. Hence, we address one main research question (RQ): Which BCT clusters are the most relevant for long-term stroke rehabilitation in a home setting in relation to treating its behavioral, cognitive, and emotional aspects?

## Methods

### Study Design

To address the RQ, we used a Delphi method, which has proven to be essential in ranking importance of BCT clusters in different contexts when large-scale data on intervention outcomes are notably rare [18,32-35].

The Delphi method was administered by email and using a link to a web-based survey tool (Qualtrics) [36]. We followed the procedure set out by Rowe et al [37] and obtained consensus after 2 rounds. The 2-round Delphi survey was used to rank the least and most important BCT clusters across the parameters outlined in the RQ. The 2 rounds were executed in 2021 between June and October. In the first round, the 16 BCT clusters defined in the behavior change technique taxonomy (BCTT; version 1) were used as the basis for the survey structure, and questions were evaluated by 12 international experts [18,33]. BCTT (version 1) was used for two main reasons: (1) it provides a basis for direct comparison with an array of prior ranking studies and hence helps to contextualize the results of this work and (2) it is widely applied in practice, making it accessible to all involved experts. In the second round, the results were iterated by 9 of the original 12 experts to reach a consensus on the ranking of the BCT clusters. The 16 BCT clusters are summarized in Textbox 1 as they were presented to the participants.

Textbox 1. **Summary of the 16 behavior change technique (BCT) clusters in behavior change technique taxonomy (version 1) as presented to participants.**

1. Goals and planning comprises BCTs that revolve around goal-setting and revision, strengthening commitment to those goals, problem-solving (by analyzing and removing factors influencing certain behavior), and action planning.
2. Feedback and monitoring comprises BCTs that revolve around monitoring (by others) or self-monitoring (yourself) the behaviors or behavior outcomes, which can—but does not have to—be combined with feedback on behavior, its outcomes, or body state (biofeedback).
3. Social support comprises BCTs that revolve around providing different types of social support to assist the person in the process of behavior change.
4. Shaping knowledge comprises BCTs that revolve around providing information on the antecedents of the behavior (including suggesting alternative explanations) and instructions on how to perform the behavior.
5. Natural consequences comprises BCTs that revolve around providing information (and occasionally also monitoring) on the health, social, environmental, and emotional consequences of the person’s behavior.
6. Comparison of behavior comprises BCTs that revolve around comparing person’s behavior to a correct behavior or others’ behavior as well as informing about what other people think about the behavior.
7. Associations comprises BCTs that revolve around providing prompts or cues as a stimulus for the behavior and gradually removing them when the goal is achieved. The cues can also be related to signaling the reward (or lack of thereof) or removing a stimulus that causes adverse effects.
8. Repetition and substitution comprises BCTs that revolve around practicing or rehearsing behaviors (sometimes in an excessive manner—overcorrection) to increase habit and skill, as well as behavior substitution with a wanted or neutral behavior, and habit formation or reversal.
9. Comparison of outcomes comprises BCTs that revolve around providing objective, credible source of information in favor or against the behavior, as well as advising comparison of pros and cons of the behavior or possible outcomes of doing (vs not doing) a given behavior.
10. Reward and threat comprises BCTs that revolve around providing a material or social reward or incentive to encourage the desired behavior. The subtle difference between a reward and incentive refers to the fact that the former is actually performed at a given time (eg, arranging for the person to receive money that would have been spent on cigarettes if and only if the smoker has not smoked for 1 month would be a material reward, while informing that the payment will be made in the future for every month a person did not smoke during pregnancy would be a material incentive). Similarly, a reward or incentive can relate to the behavior itself (eg, sticking to a diet every day for a month) or the outcome of the behavior (eg, losing 5 kg in a month). Finally, the rewards or incentives can be provided externally or by the person themselves (eg, plan to reward yourself with new clothes if you adhered to a healthy diet).
11. Regulation comprises BCTs that revolve around providing different ways to regulate and facilitate one’s behavior. That could include encouraging the use of drugs that facilitate behavior change, advising on ways of reducing negative emotions or minimizing mental resources to support behavior change, or giving paradoxical instructions to engage in the unwanted behavior with a point of reducing motivation to do it (eg, asking a smoker to smoke twice as many cigarettes a day than they usually do or tell the person to stay awake as long as possible in order to reduce insomnia).
12. Antecedents comprises BCTs that revolve around changing one’s physical or social environment to facilitate the performance of wanted behaviors or create barriers for unwanted behaviors. This can also include adding objects to the environment, advising on how to avoid or reduce exposure to specific social or physical cues that trigger unwanted behavior, or how to distract attention with other activities. It could also mean introducing body changes to facilitate behavior change (eg, strength training).
13. Identity comprises BCTs that revolve around attention to self-image related to the given behavior. It could be informing the person that their behavior may be an example to others, suggesting deliberate change of perspective on behavior (framing or reframing) to change emotions associated with the behavior or drawing attention to differences between the behavior and person’s self-image. It could also include asking the person to construct and articulate their new identity (eg, as an “ex-smoker”).
14. Scheduled consequences comprises BCTs that revolve around the planned consequences of the behavior, which could be both positive (eg, rewarding completion [or even approximation] of the behavior) or negative (eg, punishing the unwanted behavior or removing the reward).
15. Self-belief comprises BCTs that revolve around the idea of reinforcing one’s belief that they can and will successfully perform the wanted behavior and arguing against self-doubt and insecurity.
16. Covert learning comprises BCTs that revolve around imagining performing the given behavior and its consequences or observing the consequences of this behavior for others.

### Delphi Panel

For the Delphi panel, we convened 12 international experts on behavioral intervention and stroke rehabilitation based on the scientific, medical, and industrial networks of the authors, following other recent Delphi studies in this area [33,38]. Here, expert was defined as “someone with knowledge and experience on a particular subject matter” [39]. The experts were identified through the academic and professional networks of the authors (focusing on those in the fields of design and clinical practice), with eligibility criteria as follows. All experts had at least a master degree as well as multiple years of experience in practice. Further, they all had expertise in long-term behavioral design, development of interventions in this context, and familiarity with the BCTT, that is, they were able to distinguish the BCTT clusters and their implications. While these criteria led to a relatively small sample, there are several reasons for this. First, the population deeply familiar with both the full BCTT and stroke education and rehabilitation is very limited, with several of our contacts rejecting our invitation on this basis despite being relevant subject experts. This is a familiar issue for studies of BCT use; thus, our experience and sample align with an array of recent BCT-related Delphi studies [33,34]. Second, we specifically include experts focused on the development of interventions in the emerging area of behavioral design, where there are even less experienced practitioners [26]; thus, in order to maintain a balanced sample, each group was limited based on this smallest population. This subgroup was specifically included due to its growing impact on the development and implementation of health interventions [20,34]. Thus, of the 26 experts approached, we accepted a sample of 12 who agreed to participate in the study.

Overall, 12 completed the first round and 9 (75%) the second round, exceeding the minimum 70% threshold for Delphi round response [40]. Experts represented three main backgrounds involved in behavioral intervention in the health context: (1) specialists in behavioral science, design for behavior change, or psychology, who research BCTs (n=4); (2) behavioral designers working in industry actively developing health-related behavioral interventions (n=4); and (3) expert health care professionals working with patients with stroke daily (n=4). The participants were residents of Denmark (8), Germany (2), and Portugal (2). Given the heterogeneous group, this panel is in line with recommendations for a number of participants [41,42] as well as comparable studies in this area [33,38].

### Ethical Considerations

The ethics approval processes at the host research institution and national guidelines were followed. No further ethics approval was needed with reference to the Danish Research Ethics Committees that state on their website that “(...) health science questionnaire surveys and interview studies that do not involve human biological material (section 14(2) of the Danish Act on Committees) are not mandatory to report” [43].

### Procedure

#### Delphi Study First Round

The first round of the Delphi study used a web-based questionnaire distributed via Qualtrics software. The questionnaire consisted of 3 parts. In the first part, the participants were asked to provide demographic information, such as age, gender, education, professional background, type of employment, and country of residence (which in the health care context provides more information than nationality, as clinical practice and rehabilitation process differs depending on location). The second part of the questionnaire consisted of 3 open questions, where the participants could elaborate on the context of their current job or research, their understanding of “behavior change” in the context of their work, as well as what they typically consider a “long-term” change. The third and main part of the questionnaire was divided into sections corresponding to the 16 BCT clusters [18]. Each section started with a description of a given cluster (Textbox 1) as well as the specific BCTs in the cluster and examples of practical interventions using it. The description was followed by 2 questions focused specifically on how the BCTs from this cluster support stroke rehabilitation and treatment of psychological issues after stroke, including emotional, behavioral, and cognitive recovery. The questions used a standard 5-point Likert scale (rated from 1=not at all important to 5=extremely important) and are summarized below:

1. In your opinion, how important is [*BCT cluster*] in the context of rehabilitation of patients with stroke?
2. In your opinion, how important is [*BCT cluster*] for the treatment of the following psychological issues after stroke?
  - Behavioral (eg social judgment and personality changes)
  - Cognitive (eg attention, memory, or language impairment)
  - Emotional (eg, depression, anxiety, posttraumatic stress, and anger)

At the end of the questionnaire, there was an opportunity for the experts to share their impressions on the topic and any comments that arose during the study.

#### Delphi Study Second Round

After the results from the first round were analyzed, a second questionnaire was designed for each specific participant, delivered via a personalized email. This also provided feedback on the responses to the first round for participants to reconsider their prior answers. The purpose of this round was to determine consensus on only those BCT clusters where no consensus was reached in the first round. This limited the scope of the second round in order to ease participation and followed similar approaches in prior work on BCT ranking [34]. In each case, participants had the opportunity to change their prior answers in light of the first round responses or to provide additional explanations of their reasoning. The explanation of the questions and BCT clusters was the same as in the first round.

### Data Analysis: Consensus Evaluation

After both rounds of the Delphi survey were complete, the final level of consensus and ranking of the BCT clusters was determined. While the means for establishing consensus and its level in Delphi studies are debated, we apply two widely acknowledged criteria: (1) percentage consensus [44] and (2) median and IQR [33,45]. In terms of overall percentage consensus, a minimum of ≥50% is considered acceptable and provides context for the IQR evaluation [44]. When using Likert scales in Delphi studies, agreement with respect to a median score is widely evaluated with respect to IQR. Specifically, for a 5-point Likert scale, an IQR of ≤1 is suggested as indicative of a high degree of consensus [44,45]. Thus, in this study, we considered consensus to be reached when both percentage consensus was ≥50% and IQR was ≤1, that is, the majority of answers fell within 1 point on the 5-point Likert scale. The percentage consensus, median, and IQR results are summarized for all BCT clusters in Table 1 (for survey question 1) and Table 2 (for survey question 2a, b, and c).

**Table 1.** The percentage consensus and median (IQR) for all behavior change technique (BCT) clusters related to question 1 after round 2.

BCT cluster	Consensus	Median (IQR)
1. Goals and planning	67	4.0 (4.0-4.3)
2. Feedback and monitoring	42	4.0 (4.0-5.0)
3. Social support	50	4.5 (4.0-5.0)
4. Shaping knowledge	58	4.0 (3.8-4.0)
5. Natural consequences	50	3.0 (2.0-3.0)
6. Comparison of behavior	50	2.0 (2.0-3.0)
7. Associations	42	4.0 (3.0-4.0)
8. Repetition and substitution	50	4.5 (4.0-5.0)
9. Comparison of outcomes	67	4.0 (2.8-4.0)
10. Reward and threat	33	3.0 (3.0-4.0)
11. Regulation	50	3.0 (3.0-4.0)
12. Antecedents	50	4.0 (3.0-4.0)
13. Identity	42	3.5 (2.8-4.0)
14. Scheduled consequences	42	2.5 (2.0-4.0)
15. Self-belief	50	4.0 (4.0-5.0)
16. Covert learning	58	3.0 (2.8-3.0)

**Table 2.** The percentage consensus and median (IQR) for all behavior change technique (BCT) clusters related to question 2a, 2b, and 2c after round 2 (clusters are numbered for brevity).

BCT	Question 2a		Question 2b		Question 2c	
	Consensus	Median (IQR)	Consensus	Median (IQR)	Consensus	Median (IQR)
1	50	3.5 (3.0-4.0)	33	4.0 (3.0-5.0)	50	4.0 (3.0-4.0)
2	50	4.0 (3.8-4.3)	42	4.0 (3.8-5.0)	50	4.0 (3.0-4.0)
3	58	4.0 (3.5-4.0)	58	4.0 (3.0-4.0)	67	5.0 (4.0-5.0)
4	58	4.0 (3.0-4.0)	42	4.0 (3.0-4.0)	50	4.0 (2.8-4.0)
5	42	3.0 (2.0-3.3)	42	2.0 (2.0-3.0)	67	2.0 (2.0-4.0)
6	42	3.0 (2.0-4.0)	42	3.0 (1.8-3.0)	25	2.5 (1.8-3.3)
7	50	4.0 (3.0-4.0)	50	4.0 (3.0-4.0)	42	3.0 (3.0-4.0)
8	67	4.0 (4.0-4.3)	50	4.0 (4.0-5.0)	33	3.0 (2.8-4.0)
9	67	4.0 (2.0-4.0)	42	3.0 (2.0-3.3)	42	4.0 (2.8-4.0)
10	50	3.0 (2.8-3.3)	50	3.0 (2.0-3.0)	33	3.0 (2.0-4.0)
11	50	3.5 (3.0-4.0)	50	3.0 (3.0-4.0)	50	3.5 (2.8-4.0)
12	58	4.0 (3.0-4.0)	50	4.0 (2.8-4.0)	33	3.0 (2.0-4.0)
13	42	3.5 (2.8-4.0)	42	3.0 (2.8-4.0)	50	4.0 (3.8-4.3)
14	33	3.0 (2.0-4.0)	33	2.5 (1.0-3.3)	33	2.5 (2.0-4.0)
15	50	4.0 (3.8-4.3)	67	4.0 (4.0-4.0)	42	4.0 (3.8-5.0)
16	50	3.0 (2.0-3.0)	50	2.0 (2.0-3.0)	58	3.0 (2.0-3.0)

### Ranking

Once the level of consensus was determined, the mean and SD were calculated for all BCT clusters that had reached consensus. The mean was then used to rank the importance of each BCT cluster with respect to the parameters in the RQ. This follows prior work evaluating BCT importance [33].

## Results

### BCT Clusters for Long-Term Stroke Rehabilitation

In answer to which BCT clusters are the most relevant for long-term stroke rehabilitation in a home setting in general, consensus was reached for 12 of 16 BCT clusters after the second round Delphi survey. No consensus was reached for the comparison of outcomes, reward and threat, identity, and scheduled consequences (IQRs>1). The importance of the remaining 12 clusters is summarized in Table 3 based on their means, and with the 3 most and 3 least relevant highlighted in italics format. The most relevant BCT clusters were repetition and substitution (mean 4.50, SD 0.52), social support (mean 4.42, SD 0.67), feedback and monitoring (mean 4.25, SD 0.75), and self-belief (mean 4.25, SD 0.87), while the least relevant were natural consequences (mean 3.00, SD 1.04), covert learning (mean 2.83, SD 0.83), and comparison of behavior (mean 2.50, SD 0.90).

**Table 3.** Consensus importance ranking of behavior change technique (BCT) clusters for overall stroke rehabilitation.

Rank	BCT cluster	Mean (SD)
1	8. Repetition and substitution	4.50 (0.52)^a^
2	3. Social support	4.42 (0.67)^a^
3	2. Feedback and monitoring	4.25 (0.75)^a^
3	15. Self-belief	4.25 (0.87)^a^
5	1. Goals and planning	4.17 (0.58)
6	4. Shaping knowledge	3.92 (0.67)
7	12. Antecedents	3.75 (0.87)
8	7. Associations	3.58 (1.00)
9	11. Regulation	3.42 (0.79)
10	5. Natural consequences	3.00 (1.04)^b^
11	16. Covert learning	2.83 (0.83)^b^
12	6. Comparison of behavior	2.50 (0.90)^b^

a The 3 most relevant.

b The 3 least relevant.

### BCT Clusters for Behavioral, Cognitive, and Emotional Aspects of Stroke Rehabilitation

**Figure 1.**
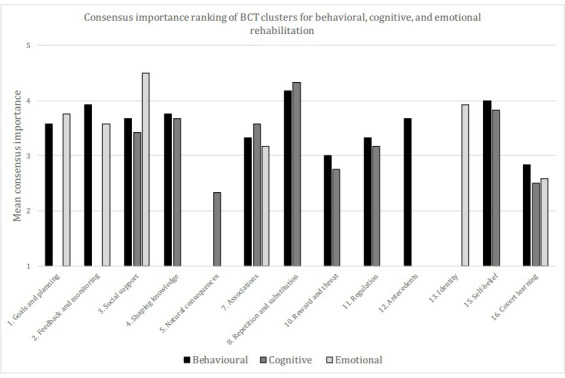
Consensus importance ranking of BCT clusters for behavioral, cognitive, and emotional rehabilitation. Clusters are displayed by number. Only those clusters that reached consensus are displayed for each aspect. BCT: behavior change technique.

In answer to our RQ regarding which BCT clusters are the most relevant for treating behavioral, cognitive, and emotional aspects, different levels of consensus were reached across the 3 aspects evaluated. For behavioral rehabilitation, 11 BCT clusters reached consensus, while for cognitive, this number was 9 and for emotional 6. The importance of the remaining clusters in each aspect is summarized in Tables 4-6 (behavioral, cognitive, and emotional, respectively). Again, the 3 most and 3 least relevant for each aspect are highlighted in italics format. For both behavioral and cognitive rehabilitation, repetition and substitution (mean 4.17, SD 0.58 and mean 4.33, SD 0.65, respectively) as well as self-belief (mean 4.00, SD 0.74 and mean 3.83, SD 0.94, respectively) were ranked as most relevant, while covert learning was consistently ranked as one of the least relevant BCT clusters across all 3 aspects. The consensus importance results for the 3 aspects are summarized in Figure 1.

**Table 4.** Consensus importance ranking of behavior change technique (BCT) clusters for behavioral rehabilitation.

Rank	BCT cluster	Mean (SD)
1	8. Repetition and substitution	4.17 (0.58)^a^
2	15. Self-belief	4.00 (0.74)^a^
3	2. Feedback and monitoring	3.92 (0.90)^a^
4	4. Shaping knowledge	3.75 (0.62)
5	3. Social support	3.67 (1.07)
5	12. Antecedents	3.67 (0.78)
7	1. Goals and planning	3.58 (0.67)
8	7. Associations	3.33 (1.23)^b^
8	11. Regulation	3.33 (0.78)^b^
10	10. Reward and threat	3.00 (0.74)^b^
11	16. Covert learning	2.83 (0.72)^b^

a The 3 most relevant.

b The 3 least relevant.

**Table 5.** Consensus importance ranking of behavior change technique (BCT) clusters for cognitive rehabilitation.

Rank	BCT cluster	Mean (SD)
1	8. Repetition and substitution	4.33 (0.65)^a^
2	15. Self-belief	3.83 (0.94)^a^
3	4. Shaping knowledge	3.67 (0.89)^a^
4	7. Associations	3.58 (1.16)
5	3. Social support	3.42 (0.79)
6	11. Regulation	3.17 (1.03)
7	10. Reward and threat	2.75 (0.87)^b^
8	16. Covert learning	2.50 (0.90)^b^
9	5. Natural consequences	2.33 (0.89)^b^

a The 3 most relevant.

b The 3 least relevant.

**Table 6.** Consensus importance ranking of behavior change technique (BCT) clusters for emotional rehabilitation.

Rank	BCT cluster	Mean (SD)
1	3. Social support	4.50 (0.80)^a^
2	13. Identity	3.92 (0.90)^a^
3	1. Goals and planning	3.75 (0.87)
4	2. Feedback and monitoring	3.58 (0.79)
5	7. Associations	3.17 (0.94)^b^
6	16. Covert learning	2.58 (0.90)^b^

a The 3 most relevant.

b The 3 least relevant.

## Discussion

### Principal Findings

Particularly in the context of the aging population, stroke rehabilitation is a high priority in health care. Yet, while the BCTs in general have received significant attention [18,33], the differences in importance introduced by long-term, multifaceted (behavioral, cognitive, and emotional) rehabilitation have not been widely studied. This study thus takes an important first step by identifying consensus on the importance of 16 BCT clusters for long-term stroke rehabilitation in general and with respect to the behavioral, cognitive, and emotional aspects of stroke rehabilitation, as detailed in the BCTs in Health Care section. Hence, our results directly address our RQ: Which BCT clusters are the most relevant for long-term stroke rehabilitation in a home setting in relation to treating its behavioral, cognitive, and emotional aspects? Specifically, they highlight 3 main insights for the prioritization of BCTs for long-term behavior change outcomes measured by adherence to treatment.

First, our study further confirms the importance of repetition and substitution and feedback and monitoring as key BCT clusters in this context. This aligns with the ranking of Vestjens et al [33], who highlight repetition and substitution as important for complex interventions in general, as well as that of O’Neill et al [46], who highlight feedback and monitoring as important for preventing smoking relapse in the long term. Further, these clusters were also ranked with social support, aligning with wider discussions of the synergy between these BCT clusters [34], as well as practical implementations via, for example, activity monitors [47]. Notably, both our ranking and the long-term focused part of our study differ substantially from the short-term focused rankings reported by O’Neill et al [46]. As such, our results contribute to a growing body of evidence that long-term behavior change emphasizes specific BCTs, which can vary substantially depending on the specific health situation or aspect of care (behavioral, cognitive, and emotional). Further, our results respond to the barriers identified by Jack et al [8] by directly highlighting BCTs that address low levels of self-efficacy, helplessness, and poor social support. In addition, they align with Campbell et al [15] and Bonnechre et al [16], and work in feedback and monitoring can provide a proxy for a physiotherapist’s presence, contact, and supervision of the patient. This provides an important insight for practitioners by pointing to potentially valuable long-term BCT clusters as well as emphasizing the need for caution when evaluating BCT rankings derived with respect to short-term interventions.

Second, our results deprioritize both shaping knowledge and natural consequences, including BCTs such as instructions, information about antecedents and health, social, or emotional consequences, and anticipated regret. This also aligns with the work of Vestjens et al [33] and complements the focus of Nieuwenhuijsen et al [31] on the need for contextual sensitivity in effective interventions. This is a notable finding, as such information-focused BCTs form a major, widely used component in many interventions in practice. For example, Guay et al [48] note that 5 of 12 studies included in a systematic review build on some combination of instructions to guide behavior change. Thus, while information is a critical component in any intervention, especially in this context, practitioners should consider these BCT clusters as supports to more direct, person-focused BCT clusters, such as feedback and monitoring or repetition and substitution.

Finally, linking to focusing on the most relevant BCTs for treating the behavioral, cognitive, and emotional aspects of rehabilitation, we note 2 important differences evident in the emotional rehabilitation results (Table 6). In this aspect, social support and identity become key BCT clusters, in contrast to Tables 3-5, as well as with the long-term ranking of O’Neill et al [46], where identity-related techniques were low ranked and social support was not included. In addition, there were much lower levels of consensus regarding the emotional aspect. This corresponds to the work of Willems et al [34], who also identified a lack of consensus with respect to “stress management or emotional control training,” while emotional aspects were also low ranked by both Vestjens et al [33] and O’Neill et al [46]. This suggests the need for further study of how the emotional aspect of stroke rehabilitation might be supported and how it differs from the more traditional focus on behavioral and cognitive aspects. The differences highlight the need for particular attention by practitioners on this aspect, as specific BCTs are required to support emotional rehabilitation, and that due to the lack of consensus in this area, careful monitoring is needed in order to evaluate their impact, and potential interaction with any behavioral or cognitive focused interventions.

### Implications and Recommendations

Our work has several implications for patients and clinicians dealing with stroke recovery. The most salient of these is acknowledging the need for dedicated attention to and support for the emotional aspects of stroke rehabilitation, distinct from behavioral and cognitive aspects. Here, there is a need to maintain a positive, progress-oriented approach, where social support and identity-forming interventions are critical. Further, there is also a more general need to ensure adequate social structures around patients during rehabilitation, going beyond the immediate behavioral and cognitive aspects of recovery. One way to support this is through the design of a social-cooperative platform that allows the patient to track their progress, such as improvements in range of motion, enables therapists to monitor and intervene in the patient’s home training, and allows relatives and other stakeholders to support the patient’s training efforts on a day-to-day basis. An example of such a system has been proposed in the recent work of Lauer-Schmaltz et al [49] on human digital twins in rehabilitation.

These results also point to several issues to consider during intervention design and clinical implementation. First, there is a need for dedicated long-term strategies to be put in place based on consideration of both long-term behavioral results and relevant BCTs but also effective design approaches for maintaining monitoring and support in the long term. Second, while information is an essential component in supporting understanding, it should not be seen as a major intervention in and of itself. Attention should be paid to coordinating multiple targeted interventions addressing each of the behavioral, cognitive, and emotional aspects. Here, care must be taken that each aspect is both individually supported and that the overall set of support is mutually reinforcing and manageable across aspects. Further, there should be provision for monitoring emotional aspects in addition to more typical behavioral and cognitive monitoring. These aspects may be addressed through, for instance, ongoing dialogue with the patients about their intrinsic motivations for rehabilitation, such as resuming previous work or re-engaging in daily activities like cooking, gardening, or sports.

### Limitations

The main limitation of this work is the use of an expert panel rather than patient or intervention data to prioritize the BCTs and to solicit patients’ perspectives on the potential impact of the results. Here, the Delphi method was used due to the general lack of access to structured data on the efficacy of BCTs in stroke rehabilitation as well as the general challenge in understanding the specific requirements of long-term behavior change. In this context, the Delphi method provides a robust means of establishing expert consensus. Second, 12 experts were involved in the Delphi survey, and while this is an acceptable number for the Delphi method and in line with prior studies, it places a focus on reaching consensus between diverse experts [38,41,42]. As such, we recruited experts from different fields of specialization, professional background, and nationalities. Thus, while further development of the panel might reveal distinctions between subgroups as in the work of Willems et al [34], the current panel provides a basis for the results reported here as well as directions for further study. Finally, while there is debate as to the required level of consensus for Delphi results, we followed prior guidance in considering BCT clusters to have reached consensus when IQR ≤1 and percentage ≥50% [35,44,50]. Notably, this highlighted a distinct lack of consensus with respect to the emotional aspect of stroke rehabilitation. This is despite this aspect being understood as important by all experts. Thus, while the included results build on prior guidance, there is a need for further study of the exact sources and nature of disagreement regarding the emotional aspect of stroke rehabilitation as well as to explore this aspect more concretely in practice via focused feasibility studies [29].

### Conclusions

This international expert panel study using a 2-round Delphi survey ranked, for the first time, the importance of 16 BCT clusters for long-term stroke rehabilitation. The process yielded several new insights highlighting differences in BCT importance between general rehabilitation and rehabilitation specifically focused on the behavioral, cognitive, and emotional aspects of stroke recovery. In particular, we identified a low level of consensus with respect to the emotional aspect but also a distinctly different BCT prioritization in comparison to the other aspects, as such, enabling more effective intervention mapping and guiding the design of multilevel rehabilitation and health promotion interventions and implementation strategies [24]. The study results provide decision support for targeted and tailored design of complex interventions for long-term behavior change and treatment adherence. This provides a first but important step toward unlocking the prioritization of BCTs for long-term intervention contexts such as stroke rehabilitation.

## Abbreviations

BCT: behavior change technique
BCTT: behavior change technique taxonomy
RQ: research question
